# Local Exome Sequences Facilitate Imputation of Less Common Variants and Increase Power of Genome Wide Association Studies

**DOI:** 10.1371/journal.pone.0068604

**Published:** 2013-07-16

**Authors:** Peter K. Joshi, James Prendergast, Ross M. Fraser, Jennifer E. Huffman, Veronique Vitart, Caroline Hayward, Ruth McQuillan, Dominik Glodzik, Ozren Polašek, Nicholas D. Hastie, Igor Rudan, Harry Campbell, Alan F. Wright, Chris S. Haley, James F. Wilson, Pau Navarro

**Affiliations:** 1 Centre for Population Health Sciences, University of Edinburgh, Edinburgh, Scotland, United Kingdom; 2 MRC Human Genetics Unit, University of Edinburgh, Edinburgh, Scotland, United Kingdom; 3 Department of Public Health, University of Split, Split, Croatia; 4 Centre for Global Health, University of Split, Split, Croatia; 5 Roslin Institute, University of Edinburgh, Scotland, United Kingdom; The Children’s Hospital of Philadelphia, United States of America

## Abstract

The analysis of less common variants in genome-wide association studies promises to elucidate complex trait genetics but is hampered by low power to reliably detect association. We show that addition of population-specific exome sequence data to global reference data allows more accurate imputation, particularly of less common SNPs (minor allele frequency 1–10%) in two very different European populations. The imputation improvement corresponds to an increase in effective sample size of 28–38%, for SNPs with a minor allele frequency in the range 1–3%.

## Introduction

Genome-wide association study (GWAS) meta-analyses routinely use genotype imputation [1]. Accurate imputation of less common variants (minor allele frequency MAF, 1–10%) may be particularly useful as commercial genotyping arrays often provide poor coverage of such variants, and imputation improves association power most for less frequent causal variants [2].

The recently released 1000 Genomes haplotypes [3] are a particularly large and dense reference panel that will be commonly used as an imputation reference panel, particularly in GWAS consortia. At the same time, theoretical studies and empirical studies using other primary reference panels, have shown that imputation accuracy in a study population can be increased by use of an additional reference panel such as whole genome or exome sequence data drawn from a subset of the population under study [2] [4] [5] [6] [7] [8] [9].

It is therefore useful to quantify the likely benefit of adding local reference data to 1000 Genomes data, particularly for less common variants, and especially if the population is genetically distant from the 1000 Genomes populations.

We used data from the CROATIA-Korcula and Orkney Complex Disease studies (ORCADES) [10] [11]. Both studies are family-based, cross-sectional community studies of the genetics of complex traits. The Croatian island of Korčula is in the Adriatic and the ORCADES study is based in the Orkney Isles in Scotland.

Genotypes obtained from the whole exome sequencing of 91/89 CROATIA-Korcula/ORCADES quality controlled samples were used to supplement the 1000 Genomes reference panel. We focused on less common (MAF 1–10%) exonic variants already in 1000 Genomes which, unlike low frequency, and rare (MAF<1%) or private variants, can be meta-analyzed in typically sized consortia.

In this paper, we therefore seek to determine if imputation accuracy can be improved by the addition of local sequences to a global reference panel.

## Methods

The ORCADES and CROATIA-Korcula studies both had ethical approval for genetic research into the basis of complex traits, approved by the appropriate committees in each country. For ORCADES the committees were the Orkney Local Research Committee and the North of Scotland Research Ethics Committee (approval Orkney: 27/2/04). For CROATIA-Korcula the committees were the Ethics Committee of the Medical School, University of Split (approval id 2181-198-03-04/10-11-0008) and the NHS Lothian (South East Scotland Research Ethics Committees; REC reference 11/AL/0222). All participants provided written informed consent.

Array genotypes were obtained from Illumina Hap370CNV array, at 319,552 SNPs for CROATIA-Korcula subjects and Illumina Omni1 array at 1,140,419 SNPs or the Illumina Human Hap300 array at 293,687 SNPs for ORCADES subjects. For ORCADES a common panel of intersecting Hap300 and Omni1 SNPs was first created. The panel for CROATIA-Korcula was then restricted to these SNPs, to ensure similar panel sizes.

Subjects to be sequenced were selected from the wider study populations that were genotyped on the Illumina Hap (370CNV/300) arrays to minimize relatedness, and thus to maximize representation of study population haplotypes. The selection was carried out by tracking the identity-by-descent sharing structure, as determined by the array genotypes using the program ANCHAP [12]. Whole exome sequences of 99/95 CROATIA-Korcula/ORCADES subjects were generated using the Agilent SureSelect All Exon 50 Mb kit and 234,746/217,015 variants were identified.

**Figure 1:**
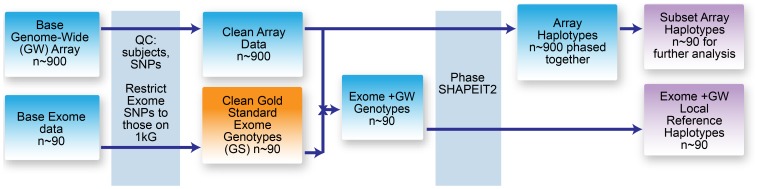
Preparation of array data and local reference panel for imputation. The genotype data were quality controlled and phased. These data were then used in further downstream analysis.

Quality control (QC) of genotyping array data, that were subsequently used for imputation, was in accordance with best practice for association studies [13] and is described in detail in Methods S1. As illustrated in Figure 1, post QC array data of 170,134/171,749 SNPs for 892/1158 Korčulan/Orcadian subjects were then pre-phased simultaneously (within each population) using SHAPEIT v1.r416 [14] [15] including the maximal pedigree structure permitted by the software (non-overlapping nuclear families) to create a phased set of study genotypes ready for imputation using IMPUTE2 v2.2.2 [16]. The simultaneous phasing of all (892/1158) study subjects allowed all these subjects’ phasing to inform the phase of the ∼100 subjects taken forward as a reference panel and for imputation.

Exome sequence data were also subjected to rigorous QC to ensure they were of high quality so that that the local reference panel we created did not have a significant number of incorrect haplotypes. Variants were called by first aligning the raw sequence data to the human hg19 reference genome using the Stampy short read aligner [17] (with BWA utilized as a pre-mapper [18]). Genotype calls were produced from the resulting alignments using GATK’s unified genotyper, following GATK’s recommended best practice for variant detection from exome sequence datasets [19]. Variants were required to have a phred-scaled quality of at least 40. Individual sample genotype calls with a phred-scaled quality less than 20 were regarded as missing. Variants that were called in less than 50% of subjects, or with a minor allele frequency of less than 0.75% were removed (hence inclusion required at least two minor alleles across samples). All variants that mapped to more than one homologous region or failed a test of Hardy-Weinberg equilibrium (HWE) with a p-value of less than 10^−4^, were also removed, leaving 99/95 CROATIA-Korcula/ORCADES subjects genotyped for 102,192/97,052 variants. The HWE test was a more stringent test than for the array data reflecting lower sample numbers and the desire to particularly ensure integrity for reference data. We restricted our analysis to individuals with exome sequences and merged the exomes with the array data for these subjects. Subjects/variants with more than 50/30 mismatching calls, between the array and sequence data were excluded, although no variants failed this test. This resulted in exomes for 93/90 subjects genotyped at 102,192/97,052 exonic SNPs being merged with array data at 170,134/171,749 SNPs for these individuals. The resulting panels had 265,929/262,513 variants which were 99.91%/99.92% concordant, based on the genotypes called on both panels for 6,397/6,285 overlapping variants. As the overall genotypic concordance could mask discrepancies for minor alleles, particularly the less common variants of interest, concordance rates for minor allele calls were calculated in the MAF 1–3% range separately. Only 1/1 (CROATIA-Korcula/ORCADES) call was discrepant on each overlapping panel, giving minor allele concordance of 99.7% in both studies for these variants.

8,150/10,964 Korčulan/Orcadian variants other than single base substitutions, for example insertions or deletions, were excluded. 119/110 conflicting map positions and individuals called at fewer than 80% of the combined SNP panel were then excluded, leaving 91/89 subjects typed across 257,633/251,439 SNPs. Our focus was on the potential to improve power in meta-analyses, so polymorphisms that were unique to each cohort were excluded. This was done by comparison to the 1000 Genomes project map and those variants not present in the 1000 Genomes reference data or with mismatches in allele codes were excluded.

The merged sequence and array data consisting of 233,195/232,096 variants for 91/89 subjects were then phased by SHAPEIT, using the recommended N_e_ of 11,418 and the default settings [14], to create reference haplotypes, as shown in the lower half of Figure 1.

**Figure 2:**
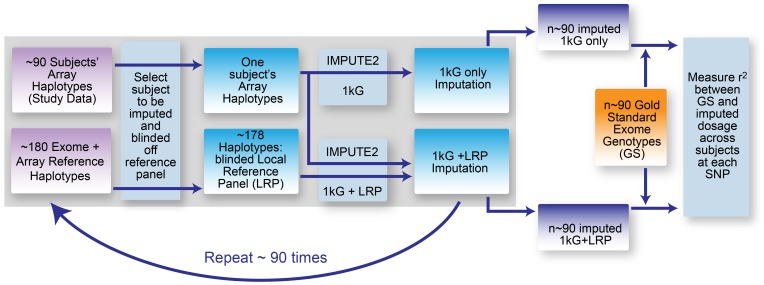
Illustration of the procedure to estimate imputation accuracy. We used a drop one-out crossvalidation approach. For the imputation step each subject was removed from the reference panel in turn, and this subject’s exome sequence SNPs were then imputed using either the 1000 Genomes reference panel alone or in conjunction with a second local reference panel. All subjects’ imputed allelic dosages were then compared with the exome sequence genotype data (“gold standard”).

Having created suitable post-QC array data and secondary reference panels, imputations were performed using genome-wide array data plus (i) 1000 Genomes haplotypes [2] alone or (ii) 1000 Genomes haplotypes together with local data as reference panels. Both imputations were then compared with known genotypes and an assessment of accuracy across all subjects was made for each SNP, as illustrated in Figure 2.

Imputation of the 91/89 subjects with and without the benefit of local reference data was carried out using IMPUTE2, using the phased reference panel option, the phased array data haplotype option, and with the software splitting the genome into chunks, which had been predetermined to be less than 5 Mb in size and avoiding crossing the centromeres. N_e_ was set to 20,000; all other settings were left at their default values. For the one panel imputation, the 1000 Genomes Phase 1 worldwide integrated variant set (March 2012 release) [3] as available on the IMPUTE2 website [16] was used. The two-panel imputation added the phased local reference data as a secondary panel (we did not use the merge panels option). All other settings for the two-panel imputation we were identical to the one panel imputation. We performed imputations for each subject with local exome data separately, with the study subject’s own haplotypes removed from the secondary reference panel so that the haplotypes of the individual to be imputed were not present in the reference data. For a given SNP, the accuracy (r^2^) of the allelic dosages imputed was measured across samples against the known exome sequence-called genotypes.

As evidenced by the genome-wide SNP array concordance data, noted above, there was close agreement between the exome sequence and independent genotyping data, indicating that the sequences were a suitable gold standard. Furthermore exome array data were also available for the CROATIA-Korcula study (although not ORCADES) and concordance between exome array and exome sequence genotypes was 99.5% and was similar across all MAF bands.

The dual use of exome sequences both as a secondary reference panel and as the gold standard to obtain imputation accuracy was considered appropriate since a subject’s imputation panel did not include their own sequence, avoiding circularity at the imputation stage.

## Results

We found a significant increase in accuracy (r^2^ of imputed against known allele dosages across samples for a given SNP) from use of a local reference panel, which was often substantial for less common variants (Table 1).

**Table 1:** Mean accuracy of imputation (r^2^ of allelic dosage across all samples for a SNP) averaged across SNPs split by Minor Allele Frequency (MAF).

MAF	1–3.2%		3.2–10%		10–32%		>32%	
Population	Korčula	Orkney	Korčula	Orkney	Korčula	Orkney	Korčula	Orkney
**N SNPs**	12132	12123	11548	10677	16243	15262	10174	9265
**r^2^ 1kG**	0.504	0.586	0.729	0.778	0.868	0.894	0.894	0.913
**r^2^ 1kG+LRP**	0.697	0.753	0.841	0.867	0.916	0.931	0.934	0.944
**Increase r^2^**	0.193	0.167	0.112	0.089	0.049	0.037	0.039	0.031
**Std dev.**	0.309	0.295	0.182	0.157	0.093	0.078	0.074	0.065
**Inc. Sample**	38%	28%	15%	11%	6%	4%	4%	1%

MAF bins increase by factors of √10, to create four exponentially increasing bins.

N SNPs: number of SNPs in MAF bin.

1kG: 1000 Genomes used as reference panel.

1kG+LRP: 1000 Genomes plus local reference panel.

Increase r^2^: Average across all SNPs in MAF bin increase in r^2^.

Std dev: The standard deviation (across SNPs) of the increase in r^2^ at each SNP.

Inc. Sample: Increase in effective sample size for GWAS.

The standard errors of mean increases are less than 0.003. All improvements in r^2^ are significantly different from zero and significantly different between MAF bands (P<0.001, two-sided t tests).

Variants with a minor allele frequency in the range 0.01–0.032 showed an increase in imputation accuracy of 0.193/0.167 (38%/28% improvement) for CROATIA-Korcula/ORCADES and 0.112/0.089 (15%/11% improvement) for variants with MAF between 0.032 and 0.100. The high accuracy of the 1000 Genomes imputation for more common variants (MAF >0.1) provided more limited scope for improvement in this category, although even for the most common variants (MAF>0.32) the accuracy of imputation increased by 0.039/0.031 (4%/3% improvement) for CROATIA-Korcula/ORCADES after adding the second (local) reference panel.

**Figure 3:**
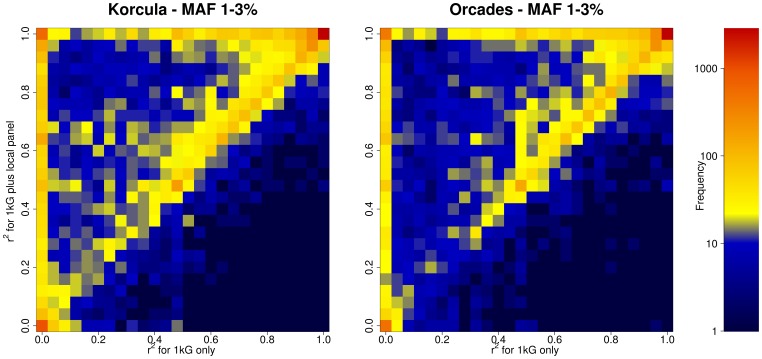
Frequency plot of imputation accuracy (r^2^) using 1000 Genomes data alone against 1000 Genomes plus a local reference panel for SNPs with Minor Allele Frequencies (MAF) of 1–3.2%.

Much of the improvements arise from SNPs that have an r^2^ close to zero with the 1000 Genomes-only imputation and which were imputed more accurately with the addition of the local panel (Figure 3). For CROATIA-Korcula/ORCADES 12%/9% of all SNPs imputed poorly (r^2^<0.2) using 1000 Genomes data alone. About one-fifth (17.1%/19.9%) of these poorly imputed SNPs imputed well (r^2^>0.8) after the addition of the local reference panel.

**Figure 4:**
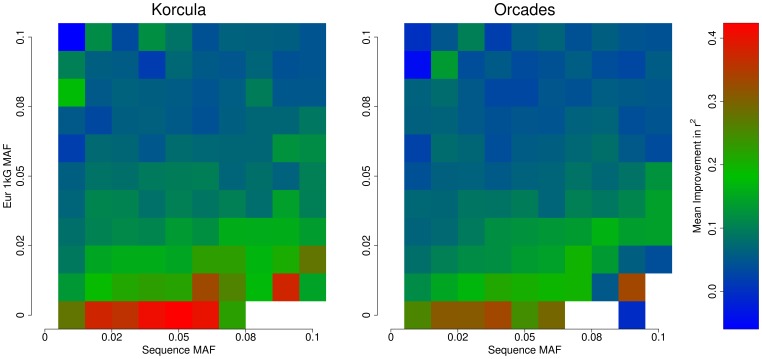
Plot of mean improvement in imputation accuracy (r^2^) for SNPs with minor allele frequency (MAF) in the range 1–10% in our exome sequence data.

SNPs that were less frequent in 1000 Genomes than in our sequences generally improved more, as illustrated in Figure 4, where areas of greater improvement are generally observed towards the right-hand side in the figure. The effect is more pronounced in Korčula and is particularly marked for variants where MAF is less than 1% on 1000 Genomes European panel.

Counts of the SNPs in each cell of Figure 4 are shown in table S1.

We also looked at r^2^ increase as a function of European 1000 Genomes MAF. As stated above, for SNPs with a MAF of 1–3.2% in our local sequences, the mean increase in r^2^ was 0.193/0.167. For these SNPs, the increase in r^2^ was 0.297/0.264 for those in the European 1000 Genomes MAF band <1%, 0.137/0.112 for MAF band 1–3.2% and 0.086/0.072 for MAF >3.2%.

## Discussion

Our results show that use of a secondary local reference panel in addition to the 1000 Genomes reference haplotype data can significantly increase the quality of imputations, particularly for less common alleles and the improvement is greater when the study population is genetically further from the populations in the reference data.

We estimated imputation accuracy using a leave-one-out cross-validation approach, in which we compared known genotypes to imputed ones using either the 1000 Genomes reference panel alone or accompanied by a panel obtained from sequence data of individuals from our study populations. Although we took care in our cross-validations to avoid circularity by using the leave-one-out approach in the imputations, for practical reasons, especially computing time, the phasing stage was done only once including all subjects (and therefore included the subject being blinded at the imputation stage). We acknowledge that this could potentially slightly inflate the reported increase in accuracy when using the second reference panel.

Imputation accuracy is not only affected by the quality and composition of the reference data used, but also by the design of the genotyping array, in particular array density and whether the array captures population specific variants [20]. A dense, locally relevant array used to genotype the study population will improve the quality of imputation compared to a less dense one, when using a global reference panel, and thus reduce the potential scope for improvement when adding local sequence data. However, where the study population’s haplotypes are distinct, due to recombination, from the reference panel population, the use of a denser array can be expected to improve the imputation but the denser array will also allow even better matching of local haplotypes, and so there should be a further benefit from use of a local secondary reference panel.

Consistent with this hypothesis, the accuracy of base imputations using only the 1000 Genomes reference panel was greater for ORCADES than CROATIA-Korcula, presumably due to the greater proximity of Orkney to subjects in the 1000 Genomes reference panel. Twenty three Orcadians, 77 mainland British and 100 of northern European ancestry individuals are present in the 1000 Genomes data, and principal component analysis shows that Balkan populations (such as Korčula) are more distant from the nearest subjects in 1000 Genomes (Tuscans, from central Italy, N = 100), than the variation observed within the British Isles [3] [21]. This suggests to us that, as might be expected, imputation improvement due to addition of local data will be most marked for populations genetically distant from 1000 Genomes samples. Whilst part of the benefit arises from including reference data with allele frequencies closer to the study population, the capture of representative local haplotypes further contributes to the increase in imputation accuracy, and this latter effect will be more marked, or at least require fewer local subject to be sequenced, in isolated populations, where fewer distinct haplotypes will be segregating.

Similarly the much greater improvements in accuracy for SNPs where the MAF is greater in our sequences than 1000 genomes, perhaps not surprisingly, shows that local sequences will add value to imputations in regions of the genome where drift, or other forces, have created a distinct genetic structure.

Comparing these results with those of other researchers who have examined the benefits of study specific reference panels, often using 1000 Genomes like us or HapMap [22] as primary panels, whilst illuminating, is not straightforward. Inevitably, different types and sizes of reference panels are used, as well as different genotyping arrays for the subjects whose genotypes are to be imputed. This is further complicated by different study protocols and differing genetic structure of the study populations. With these caveats, our results of an r^2^ of 0.70–0.75 from 90 reference panel subjects in addition to 1000 Genomes seem consistent with those of Liu et al [9] and Auer et al [8], for MAF 1–3%. Neither of these studies used a global reference panel, but Liu et al, in their verification step, attained an r^2^ of around 80% with ∼2,000 subjects on their (array data) reference panel with unfiltered results, whilst Auer et al obtained an r^2^ of 82% with 761 exome reference panel subjects, albeit filtering out lower quality results, using an Rsq threshold of 0.8, where Rsq is equivalent to the squared correlation between nearby imputed and genotyped SNPs [8]. Furthermore the latter study demonstrated that the use of exome imputation can reveal genome-wide significant associations, not discovered by conventional genotyping arrays, as did the study by Holm et al [23], who were able to discern a local rare variant causing sick sinus syndrome, in a large Icelandic study, due to the benefit of adding 87 whole genome sequences to the reference data for their imputation.

Many aspects of our study were similar to a study by Surakka et al [6]. Their Finnish study used 200 (CEU+TSI) HapMap [22] subjects as their primary reference panel and added 81 local subjects genotyped by a genome wide array. For alleles with a MAF <5%, they obtained a median r^2^ of 90% for their global panel only imputation rising to 94% after the addition of their local panel. In our study, we report mean r^2^, but our median r^2^ was 0.77/0.83 rising to 0.88/0.92 after adding the local reference panel for CROATIA-Korcula/ORCADES for a MAF bucket 3–5%. The choice of a 3–5% MAF is intended to correspond to typical array SNPs with MAF<5%. Our results therefore appear consistent with the results of Surakka et al. despite the differences in study design. The study by Uricchio et al [7] obtained much higher mean r^2^ (99%), and the technique used for imputation, identifying runs of identity-by-descent (IBD), should be particularly accurate, but its application is restricted to populations which share long haplotypes to a much greater extent than is common in most genetic studies, and we therefore feel our strategy of using 1000 Genomes reference data and adding sequence data from a subset of one’s own study subjects is a good practical way forward for many studies.

A proportionate increase in r^2^ has the same effect on power as a corresponding increase in study size [24] so the use of high quality sequence data has the potential to provide substantially greater power in GWAS studies for less common variants, particularly those very poorly imputed using 1000 Genomes alone but well imputed with the addition of local exome sequence data.

Our study focused on the exome, but the results should extend to any other genomic region of interest. Moreover, the similar results obtained in our study for two independent populations suggest that corresponding benefits will be found in other studies.

The meta-analysis of multiple populations imputed using local exome sequence data will likely identify new SNP associations. However the amount of variance explained by less common variants individually is likely to be small and will make their detection challenging. This will put increasing emphasis on the use of analytical methods that consider jointly groups of variants, be it gene [25], regional heritability [26] or network based analyses [27]. Such analyses can also incorporate the potentially valuable information provided by variants private to individual populations including the 24,438/19,343 variants identified by the exome sequencing of the CROATIA-Korcula and ORCADES samples that are not present in 1000 Genomes and hence we have not considered here.

Given the cost and significant practical difficulties in subject recruitment, sequencing a subset of cohort members, for either part or all of the genome, and using these results for imputation will provide significant added value to association studies.

## Supporting Information

Methods S1 **Quality Control of Array Data.**
(DOCX)

Table S1 **Counts of SNPs in each cell underpinning** **Figure 4** **.**
(DOCX)
