# Supplementary material for: Local Exome Sequences Facilitate Imputation of Less Common Variants and Increase Power of Genome Wide Association Studies
Source: PLoS One. 2013 Jul 16;8(7):e68604. doi: 10.1371/journal.pone.0068604 (PMC3712964; doi:10.1371/journal.pone.0068604)
Supplement: Methods S1 — Quality Control of Array Data. (DOCX) [file pone.0068604.s001.docx]

Supplementary Method – S1

Quality Control (QC) of Array Data

The Korčulan/Orcadian (99/95) exome sequenced subjects’ array genotype data was quality controlled alongside the other 801/1069 samples available in each population. 892 Korčulan subjects were genotyped using the Illumina Hap370CNV array, at 319,552 SNPs. Orcadian subjects were genotyped on the Illumina Omni1 array at 1,140,419 SNPs or the Illumina HumanHap300 array at 293,687 SNPs. An intersecting panel of 178,477 SNPs was obtained for 1159 Orcadian subjects.

Individuals that failed to genotype at more than 3% of SNPs were excluded, and SNPs that failed to genotype in more than 10% of samples, or failed a test for Hardy-Weinberg equilibrium (p-value=10^-6^) were excluded. In creating the reference panel, our aim was to create a robust and diverse local panel, so genetic outliers were not excluded, provided that other QC thresholds were satisfactory. Our array data was remapped using LiftOver[^1^](#_ENREF_1) from NCBI build 36 to build 37.3, to match the exome and 1,000 Genomes data - 9,181/4,607 SNPs were not successfully mapped to the newer build.

1,538/2,115 SNPs and 0/1 samples failed QC, resulting in 892/1158 samples genotyped at 308,833/171,749 SNPs for CROATIA-Korcula/ORCADES. To avoid possible discrepancies associated with a different size of SNP panel, the larger CROATIA-Korcula panel was then restricted to those intersecting SNPs on the post-QC Orkney panel (but not vice-versa).

1. Hinrichs, A.S., Karolchik, D., Baertsch, R., Barber, G.P., Bejerano, G., Clawson, H., Diekhans, M., Furey, T.S., Harte, R.A., Hsu, F., et al. (2006). The UCSC Genome Browser Database: update 2006. Nucleic Acids Res 34, D590-598.
