# Supplementary material for: Local Exome Sequences Facilitate Imputation of Less Common Variants and Increase Power of Genome Wide Association Studies
Source: PLoS One. 2013 Jul 16;8(7):e68604. doi: 10.1371/journal.pone.0068604 (PMC3712964; doi:10.1371/journal.pone.0068604)
Supplement: Table S1 — Counts of SNPs in each cell underpinning Figure 4 . (DOCX) [file pone.0068604.s002.docx]

# Supplementary Table S2

# Counts of SNPs in each cell underpinning Figure 4. Each cell shows the count of SNPs for the MAF intervals defined by the intersection of row and column headers, where row and column headers represent the lower bounds of those intervals.

| Local Exome Sequence MAF – CROATIA-Korcula | | | | | | | | | | |
| --- | --- | --- | --- | --- | --- | --- | --- | --- | --- | --- |
|  | 0.01 | 0.02 | 0.03 | 0.04 | 0.05 | 0.06 | 0.07 | 0.08 | 0.09 | 0.1 |
| 1kG MAF |  |  |  |  |  |  |  |  |  |  |
| 0.10 | 2 | 5 | 6 | 19 | 40 | 88 | 114 | 107 | 91 | 130 |
| 0.09 | 4 | 9 | 24 | 26 | 57 | 91 | 111 | 143 | 102 | 107 |
| 0.08 | 3 | 13 | 44 | 49 | 152 | 159 | 139 | 146 | 84 | 106 |
| 0.07 | 10 | 38 | 78 | 86 | 196 | 170 | 151 | 142 | 84 | 90 |
| 0.06 | 25 | 108 | 144 | 128 | 243 | 199 | 169 | 134 | 80 | 72 |
| 0.05 | 79 | 179 | 225 | 180 | 303 | 281 | 168 | 98 | 62 | 33 |
| 0.04 | 205 | 300 | 306 | 202 | 274 | 193 | 117 | 68 | 26 | 23 |
| 0.03 | 560 | 622 | 514 | 288 | 260 | 187 | 67 | 44 | 14 | 12 |
| 0.02 | 955 | 817 | 456 | 178 | 194 | 81 | 28 | 11 | 4 | 2 |
| 0.01 | 2097 | 1068 | 523 | 178 | 100 | 39 | 9 | 3 | 3 | 0 |
| 0.00 | 3933 | 934 | 210 | 52 | 30 | 8 | 0 | 0 | 1 | 0 |

| Local Exome Sequence MAF - ORCADES | | | | | | | | | | |
| --- | --- | --- | --- | --- | --- | --- | --- | --- | --- | --- |
|  | 0.01 | 0.02 | 0.03 | 0.04 | 0.05 | 0.06 | 0.07 | 0.08 | 0.09 | 0.1 |
| 1kG MAF |  |  |  |  |  |  |  |  |  |  |
| 0.10 | 1 | 7 | 17 | 22 | 38 | 71 | 91 | 119 | 103 | 121 |
| 0.09 | 3 | 7 | 15 | 56 | 60 | 80 | 111 | 107 | 122 | 158 |
| 0.08 | 3 | 24 | 49 | 66 | 122 | 140 | 164 | 156 | 147 | 153 |
| 0.07 | 8 | 65 | 86 | 101 | 134 | 179 | 178 | 175 | 114 | 90 |
| 0.06 | 45 | 111 | 133 | 144 | 170 | 217 | 189 | 153 | 110 | 68 |
| 0.05 | 105 | 184 | 256 | 203 | 242 | 227 | 217 | 146 | 96 | 55 |
| 0.04 | 195 | 300 | 319 | 246 | 231 | 220 | 125 | 86 | 51 | 28 |
| 0.03 | 544 | 614 | 489 | 380 | 255 | 157 | 92 | 65 | 38 | 23 |
| 0.02 | 1005 | 740 | 481 | 266 | 170 | 103 | 43 | 31 | 14 | 8 |
| 0.01 | 2075 | 1004 | 499 | 251 | 137 | 61 | 22 | 15 | 4 | 3 |
| 0.00 | 3880 | 1039 | 296 | 116 | 41 | 22 | 7 | 0 | 0 | 0 |

MAF: Minor allele frequency

1kG: 1000 Genomes
